# USP7 deubiquitinates and stabilizes EZH2 in prostate cancer cells

**DOI:** 10.1590/1678-4685-GMB-2019-0338

**Published:** 2020-05-20

**Authors:** Jae Eun Lee, Chan Mi Park, Jung Hwa Kim

**Affiliations:** 1 Inha University, Department of Biological Sciences, Incheon 22212, South Korea.

**Keywords:** USP7, EZH2, P5091, EZH2 inhibitors, prostate cancer cells

## Abstract

Regulation of target proteins by the ubiquitin-proteasome system (UPS) is common in a wide range of cellular events, including transcriptional regulation, cell cycle progression, differentiation, and tumorigenesis. Ubiquitin-specific protease 7 (USP7) has been implicated in tumor development and metastasis in various malignancies through the regulation of target protein stability. In this study, we found that the enhancer of zeste homolog 2 (EZH2), which catalyzes the methylation at lysine 27 of histone H3, is a target of USP7 and is stabilized by USP7-mediated deubiquitination. In prostate cancer cells, the transcriptional repression function of EZH2 was inhibited by USP7-knockdown. Furthermore, ectopic introduction of EZH2 restored the cell migration, invasion, and sphere-forming potential of prostate cancer cells, which had been decreased by USP7-knockdown. Moreover, combined treatment with the USP7-specific inhibitor P5091 and EZH2 inhibitors, such as GSK126, EPZ6438, and DZNep, induced synergistic inhibitory effects on cell migration, invasion, and sphere-forming potential in prostate cancer cells. Collectively, our findings revealed that the promotion of the malignancy-associated characteristics of prostate cancer cells by USP7 was in part due to EZH2 stabilization. Thus, we suggest that simultaneous treatment with a USP7 inhibitor and an EZH2 inhibitor could be a rational strategy for treating EZH2-dependent cancers.

## Introduction

The ubiquitin-proteasome system (UPS) is involved in many biological processes, including cell cycle progression, signal transduction, immune response, stress response, and tumorigenesis (Hershko and Ciechanover, 1998). Ubiquitin (Ub) is conjugated to target proteins by the serial actions of Ub-activating enzyme E1, Ub-conjugating enzyme E2, and Ub ligase E3. The properties of target proteins, such as stability, function, and subcellular localization, are changed by ubiquitination. Deubiquitination, the cleavage of Ub moieties, is mediated by deubiquitinating enzymes (DUBs) (Kim *et al.*, 2003), and this process reverses the functional changes caused by ubiquitination. Thus, the DUBs are one of the major regulatory axes of UPS and are important players in many cellular processes, including cancer development (Hanpude *et al.*, 2015; Wei *et al.*, 2015).

Various epigenetic modifications of histone proteins, which are performed by distinct histone-modifying enzymes, modulate gene expression by altering chromatin structure or attracting histone modifiers. Previous studies have indicated that the dysregulation of histone-modifying enzymes occurs frequently in many human cancers (Pfister and Ashworth, 2017). Dysregulated or mutated histone-modifying enzymes disturb the normal epigenetic pattern of histones and produce a genetic environment that is favorable for tumor development and progression.

Enhancer of zeste homolog 2 (EZH2), a member of polycomb repressive complex 2 (PRC2), trimethylates lysine 27 of histone H3 (H3K27me3) and regulates several genes involved in tumor development (Chang and Hung, 2012; Wen *et al.*, 2017). The crucial involvement of EZH2 in carcinogenesis has been reported in various types of tumors, thus making it an attractive therapeutic target for cancer treatment (Wen *et al.*, 2017). Mutant forms and abnormal expression patterns of EZH2 have been detected in several cancers and are frequently associated with tumor progression and poor prognosis (Sauvageau and Sauvageau, 2010; Chang and Hung, 2012). Therefore, the regulatory mechanism involved in the expression, activity, and stability of EZH2 can be a suitable candidate for cancer-targeted therapy. The regulation of EZH2 protein levels by UPS has been previously reported. EZH2 ubiquitination by several E3 ubiquitin ligases and subsequent proteasome-mediated degradation have been described in a few tissues and distinct physiological conditions (Wang *et al.*, 2018). Further, DUBs, including USP21 and ZRANB1, targeting EZH2 function and protein stability have been studied (Chen *et al.*, 2017; Zhang *et al.*, 2018). Recently, we reported the deubiquitinating activity of USP44 and the subsequent stabilization of EZH2 protein in prostate cancer cells (Park *et al.*, 2019).

The ubiquitin-specific protease USP7 regulates many proteins involved in DNA damage repair, cell growth, and apoptosis, including p53, HDM2, FOXO4, PTEN, CCDC6, and AR (Bhattacharya *et al.*, 2018). Recently, the list of USP7 target proteins has been updated to include the proteins involved in chromatin remodeling and epigenetic regulation, such as H2B, RING1B, BMI1, UHRF1, DNMT1, LSD1, and SUV39H1 (Bhattacharya *et al.*, 2018). As many target proteins of USP7 have important roles in cancer development, USP7 has been regarded as a promising therapeutic target.

To further investigate the role of USP7 in epigenetic regulation, we tested the interaction between several histone-modifying enzymes and USP7. In this report, we determined that the positive effects of USP7 on cell migration, invasion, and sphere-forming potential are partly due to EZH2 protein stabilization in prostate cancer cells. Furthermore, the USP7-specific inhibitor P5091 augmented the inhibition of cell migration, invasion, and sphere-forming abilities by EZH2 inhibitors in prostate cancer cells. Collectively, our findings suggest that combined treatment with a USP7 inhibitor and an EZH2 inhibitor may be a rational strategy for the treatment of EZH2-dependent cancers.

## Materials and Methods

### Cell lines and culture

HEK293T, PC3, and DU145 cells were obtained from the American Type Culture Collection (ATCC). PC3 and HEK293T cells were cultured in DMEM, and DU145 was cultured in RPMI 1640. All media was supplemented with 10% FBS containing antibiotic-antimycotic solution (0.25 μg/mL amphotericin B, 100 μg/mL streptomycin, and 100 U/mL penicillin). Using PCR, all cells were tested regularly for mycoplasma contamination. All cells were maintained in an incubator at 37 °C in a humidified atmosphere containing 5% CO_2_.

### Antibodies, chemicals, and immunoblotting

The following antibodies and drugs were used: USP7 (Santa Cruz Biotechnology, sc-137008), EZH2 (Cell Signaling, #4905), H3K27me3 (EDM Millipore, #07-449), GFP (Santa Cruz Biotechnology, sc-9996), Xpress (Invitrogen, P/N 46-0528), Flag M2 (Sigma-Aldrich, F3165), HA (Covance, MMS-101R), β-actin (Sigma-Aldrich, A1978), P5091 (Selleckchem, S7132), GSK126 (Selleckchem, S7061), EPZ6438 (Selleckchem, S7128), and DZNep (Cayman, 13828). For the immunoblotting assays, protein samples were separated by SDS/PAGE and transferred to PVDF membranes (Millipore). After blocking with 5% non-fat milk in TBS with 0.1% Triton X-100 (TBST), corresponding primary antibodies were used to probe the proteins of interest. HRP-conjugated secondary antibodies (Jackson Laboratories) and an enhanced chemiluminescence system were used for protein signal detection.

### Generation of stable USP7-knockdown cells

The target sequences of the USP7 small hairpin RNA were 5’-AAAGTCGTTCAGTCGTCGTAT-3’ (shRNA1) (van der Horst *et al.*, 2006) and 5’-AAAGTCATTTGGGT GGGAAAC-3’ (shRNA3) (Lim *et al.*, 2015). USP7 shRNA was cloned into pMSCVpuro vector. The resulting pMSCVpuro-USP7 shRNA, VSV-G, and MLV vectors were co-transfected into HEK 293 cells to generate retroviruses. Forty-eight hours later, filtered retroviruses were mixed with polybrene and added to PC3 or DU145 cells for infection. Virus-infected cells were then maintained in puromycin (5 μg/mL) for selection. USP7 shRNA stable cells were isolated after 2 weeks following selection. The pMSCVpuro empty vector was used as a control (CTL) for the knockdown assays.

### Real-time RT-PCR

After extracting total RNA using Trizol reagent, the reverse transcription reaction was performed using oligo (dT) primers and RevertAid reverse transcriptase. Semiquantitative real-time RT-PCR was performed to detect the relative mRNA abundance using SYBR green and ABI 7300. To calculate the mRNA quantity of specific genes, we used the ΔΔCt method; the *GAPDH* gene was used for normalization. The following primer pairs were used: *USP7* 5’-CGCTGGGGAACATGGCTTAC-3’ and 5’- TTGGTCCGTCTGAGGGTCAT-3’; the *EZH2*, *E-cadherin*, *ADRB2*, *SLIT2*, and *DAB2IP* primers were described previously (Park *et al.*, 2019). All the measurements were performed thrice.

### *In vivo* ubiquitination assay

The cells were treated with MG132 (10 μM) for 12 h before harvesting. Forty-eight hours after transfection, the cells were lysed in lysis buffer (25 mM Tris-HCl [pH 7.8], 150 mM NaCl, 1 mM EDTA, 0.1% NP-40, and 0.25% SDS). The lysed cells were boiled for 15 min. The clarified extracts were immunoprecipitated with anti-HA antibody. After denaturation, the samples were subjected to SDS/PAGE and immunoblotted.

### Cell proliferation assay

PC3 and DU145 cells were plated at a density of 5 × 10^4^ cells per well in six-well plates in duplicate. After 24 h, which was expressed as D0, the cells were treated with EZH2 inhibitors either in the presence or absence of P5091 for 4 days. At the indicated time points, viable cells were counted using the trypan blue-exclusion assay.

### Wound healing assay

For the wound healing assay, 3 × 10^5^ PC3 stable cells or 2 × 10^5^ DU145 stable cells per well were seeded in six-well dishes and grown to confluency. The cell monolayers were scraped using a sterile yellow micropipette tip to create a denuded area. Cells were washed with PBS to remove the detached cells and supplemented with serum-free culture medium. Wound closure was monitored and photographed using a light microscope (IX51, Olympus) at 50X magnification. The percentage of the area covered by the migrated cells at t = 22 h was calculated by normalizing to the uncovered area att=0h using ImageJ software.

### Transwell cell migration and invasion assay

For the cell migration and invasion assay, a Transwell chamber with 8-μm pore size polycarbonate membrane filters (Corning) was used. The membrane was coated with Matrigel (Corning) in the invasion experiment but not in the migration experiment. In this assay, 1 × 10^4^ PC3 or DU145 stable cells suspended in serum-free medium were loaded into the upper chamber, and the lower chamber was filled with medium containing 15% FBS. After incubation at 37 °C for 22 h, the cells that had migrated or invaded to the lower surface of the filter were fixed with 100% methanol and stained with 0.5% crystal violet solution. The number of cells that had migrated or invaded to the membrane filter was counted using a light microscope.

### Sphere formation assay

For the sphere formation assay, PC3 or DU145 cells were dissociated into single cells and seeded in 96-well Ultra-low Attachment plates (Corning) at a density of 100 cells/well and cultured in serum-free DMEM/F12K medium (Welgene) supplemented with 4 μg/mL insulin, B27, and 20 ng/mL EGF and bFGF. After 7 days, the sphere-forming ability was assessed as the number of spheres with a diameter exceeding 100 μm under a microscope at 200X magnification.

## Results

### EZH2 interacts with USP7

**Figure 1:**
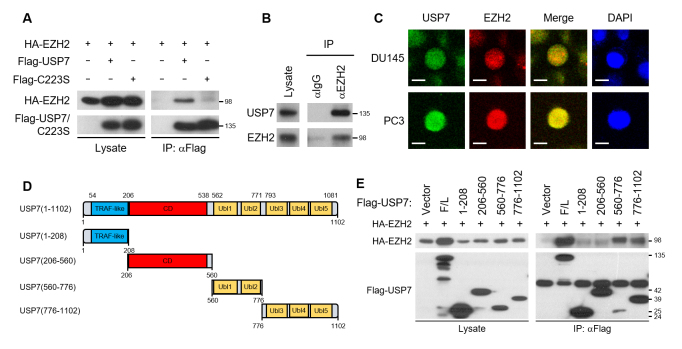
USP7 interacts with EZH2. (A) HA-EZH2 was expressed in HEK293T cells with Flag-USP7 or Flag-USP7 C223S. Cell lysates were immunoprecipitated with anti-Flag antibody and immunoblotted with anti-Flag or anti-HA antibody. (B) Endogenous interaction of USP7 and EZH2. DU145 cell lysates were immunoprecipitated with normal IgG or anti-EZH2 antibody and immunoblotted with anti-USP7 or anti-EZH2 antibody. (C) Immunofluorescence staining of USP7 and EZH2 in DU145 or PC3 cells. USP7 was stained green and EZH2 was stained red. The blue signal represents nuclear DNA stained by DAPI. The bar indicates 10 μm. (D) Schematic representation of the USP7 deletion mutants. TRAF-like, TNF receptor-associated factor (TRAF)-like N-terminal domain; CD, catalytic domain; Ubl, Ub-like domain. (E) HA-EZH2 was expressed in HEK293T cells in combination with each of the Flag-tagged USP7 deletion mutants. Cell lysates were immunoprecipitated with anti-Flag antibody and immunoblotted to detect HA-EZH2 and Flag-tagged USP7 deletion mutants.

To investigate the regulation of histone-modifying enzymes by USP7, we tested the interaction between several histone-modifying enzymes and USP7 using the immunoprecipitation assay and found that EZH2 interacts with USP7 (Figure S1A and B). The interaction of USP7 with EZH2 has been previously reported by de Bie *et al.* (2010); they demonstrated the interaction of USP7 with Polycomb group (PcG) proteins, including RING1B, RING1A, SUZ12, EZH2, and BMI1, using the GST pulldown assay. Through the immunoprecipitation assay, we found that GLP1 and LSD1, in addition to EZH2, also interacted with USP7 (Figure S1B). Among these three, the EZH2 protein expression was increased the most by USP7 overexpression (Figure S1C). Thus, subsequently, we focused on analyzing the relationship between USP7 and EZH2. The interaction between USP7 and EZH2 was detected in cells expressing the wild-type USP7 but not in those expressing the USP7 active site mutant (C223S); in the latter, the deubiquitinating activity was disabled due to the substitution of cysteine 223 to serine (Figure 1A and Figure S5A). We confirmed the endogenous interaction between EZH2 and USP7 in the metastatic prostate cancer cell line DU145 (Figure 1B). We next verified the co-localization of USP7 and EZH2 in the nucleus by immunocytochemistry (Figure 1C). To further determine the interaction region of USP7 with EZH2, we constructed various deletion mutants of USP7 (Figure 1D and Figure S5B-E). Immunoprecipitation assays showed that the full length USP7, Ub-like (Ubl) domain12, and Ubl domain345 at the C-terminal domain (CTD) interacted with EZH2 (Figure 1E). Several proteins, including GMPS, ICP0, FOXO4, and DNMT1, have been known to interact with the CTD of USP7 (Zhou *et al.*, 2018).

### EZH2 protein is stabilized by USP7-mediated deubiquitination

**Figure 2:**
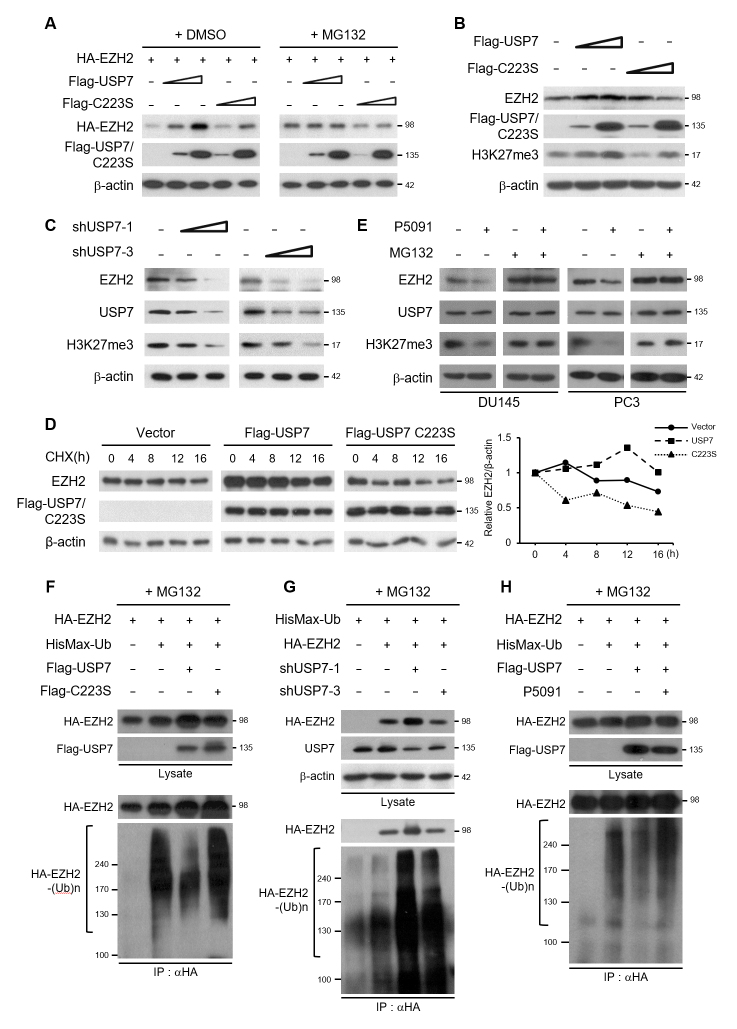
EZH2 is stabilized through USP7-mediated deubiquitination. (A) HA-EZH2 was expressed in HEK293T cells with Flag-USP7 or Flag-USP7 C223S. Cells were treated with DMSO or 10 μM MG132 for 12 h before harvesting. Cell lysates were immunoblotted with anti-HA or anti-Flag antibody. (B) HEK293T cells were transfected with increasing amounts of Flag-USP7 or Flag-USP7 C223S. Cell lysates were immunoblotted to detect Flag-tagged USP7, EZH2, or H3K27me3. (C) HEK293T cells were transfected with increasing amounts of USP7 shRNAs. Cell lysates were immunoblotted to detect EZH2, USP7, or H3K27me3. (D) HEK293T cells expressing empty vector (control) or Flag-USP7/USP7 C223S were treated with cycloheximide (CHX) for the indicated durations. Cell lysates were immunoblotted with anti-EZH2 or anti-Flag antibody. The line graph represents the relative protein levels of EZH2 normalized to those of β-actin using a densitometer and expressed as relative intensity compared to the non-treated control. (E) DU145 or PC3 cells were treated with P5091 (12.5 μM) for 24 h and MG132 (10 μM) for 12 h. Cell lysates were immunoblotted to detect EZH2, USP7, or H3K27me3. (F), (G), and (H) HEK293T cells were transfected as indicated and treated with MG132 for 12 h before harvesting. Cell lysates were then subjected to immunoprecipitation with HA antibody followed by immunoblotting with anti-HA or anti-Xpress antibody.

To characterize the functional link between EZH2 and USP7, we assessed whether the stability of EZH2 protein is affected by USP7. Transfected EZH2 protein was stabilized in proportion to the amount of USP7. However, the active site mutant form of USP7 did not stabilize EZH2 (Figure 2A). Furthermore, when the cells were treated with MG132, a 26S proteasome inhibitor, no significant difference in EZH2 stabilization was detected between the cells expressing wild-type USP7 and those expressing the USP7 C223S mutant. Endogenous EZH2 protein was also stabilized by USP7 depending on its deubiquitinating activity. The level of H3K27me3, the catalytic target of EZH2, increased with the stabilization of EZH2 by USP7 overexpression (Figure 2B). Conversely, knockdown of USP7 by USP7-specific shRNA destabilized the endogenous EZH2 and resulted in a decrease in the H3K27me3 levels (Figure 2C). These results indicate that USP7 stabilizes proteasome-dependent EZH2 degradation.

To further confirm the stabilization of EZH2 by USP7, we determined EZH2 protein levels after inhibiting protein synthesis by cycloheximide (CHX) treatment in the presence of wild-type USP7 or USP7 C223S in HEK293T cells. The protein stability of EZH2 was strongly increased by wild-type USP7 overexpression compared to the control (Figure 2D). To further validate our findings, we tested whether USP7-specific inhibitor suppresses USP7-mediated stabilization of EZH2 protein. Treatment with the specific USP7 inhibitor P5091 reduced EZH2 protein level in DU145 and PC3 cells. In the presence of MG132, EZH2 protein was not destabilized with P5091 treatment, suggesting that USP7 inhibitor promotes the proteasome-dependent degradation of EZH2 (Figure 2E). It was also confirmed that destabilization of EZH2 by P5091 treatment leads to a decrease in the H3K27me3 levels (Figure 2E). Thus, treatment with USP7 inhibitor P5091 promoted EZH2 destabilization, similar to the effect observed after USP7-knockdown.

To further address the regulatory mechanism underlying EZH2 protein stabilization by USP7, we examined whether EZH2 is a deubiquitination target of USP7. Figure 2F shows that the ubiquitinated form of EZH2 was markedly reduced after USP7 overexpression. On the contrary, USP7-knockdown increased the ubiquitinated form of EZH2 (Figure 2G). The ability of USP7 to remove Ub from EZH2 was inhibited by treatment with P5091 (Figure 2H). These results indicated that USP7 stabilized EZH2 protein through deubiquitination of EZH2.

### Oncogenic EZH2 mutants are stabilized by USP7

**Figure 3:**
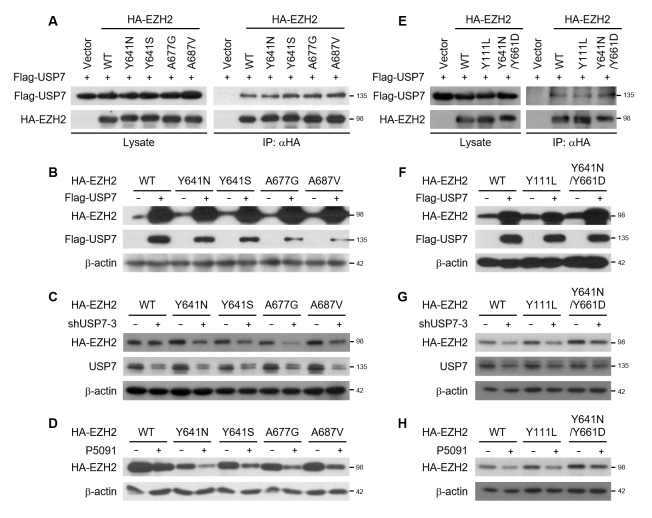
USP7 stabilizes oncogenic EZH2 mutants. (A) Flag-USP7 was expressed in HEK293T cells with HA-tagged gain-of-function EZH2 mutants. Cell lysates were subjected to immunoprecipitation with HA antibody and immunoblotted with anti-Flag or anti-HA antibody. (B) HEK293T cells were transfected with Flag-USP7 and HA-tagged gain-of-function EZH2 mutants. Cell lysates were immunoblotted with anti-Flag or anti-HA antibody. (C) HEK293T cells were transfected with USP7 shRNA and HA-tagged gain-of-function EZH2 mutants. Cell lysates were immunoblotted with anti-HA or anti-USP7 antibody. (D) HA-tagged gain-of-function EZH2 mutants were expressed in HEK293T cells, which were then treated with P5091 (12.5 μM) for 24 h before harvesting. Cell lysates were immunoblotted with anti-HA antibody. (E) Flag-USP7 was expressed in HEK293T cells with HA-tagged therapy-resistant EZH2 mutants. Cell lysates were subjected to immunoprecipitation with HA antibody and immunoblotted with anti-Flag or anti-HA antibody. (F) HEK293T cells were transfected with Flag-USP7 and HA-tagged therapy-resistant EZH2 mutants. Cell lysates were subjected to immunoblotting with anti-HA or anti-Flag antibody. (G) HEK293T cells were transfected with USP7 shRNA and HA-tagged therapy-resistant EZH2 mutants. Cell lysates were immunoblotted with anti-HA or anti-USP7 antibody. (H) HEK293T cells were transfected with HA-tagged therapy-resistant EZH2 mutants and treated with P5091 (12.5 μM) for 24 h before harvesting. Cell lysates were immunoblotted with anti-HA antibody.

Next, we examined whether the protein stability of EZH2 mutants is also regulated by USP7. In several lymphomas, gain-of-function (GOF) EZH2 mutations targeting the catalytic SET domain (Y641H, Y641S, Y641N, Y641F, A687V, or A677V) have been identified (Morin *et al.*, 2010; Majer *et al.*, 2012; McCabe *et al.*, 2012a). These GOF EZH2 mutations promote H3K27 hypertrimethylation and contribute to lymphoma pathogenesis. USP7 interacted with all the GOF EZH2 mutants tested in this study (Figure 3A and Figure S6). The protein levels of each EZH2 mutant were increased by USP7 overexpression (Figure 3B). In contrast, USP7-knockdown and P5091 treatment reduced the protein levels of all the EZH2 GOF mutants (Figure 3C and D). One major issue that limits the long-term effects of targeted cancer therapy is acquired resistance. Two novel EZH2 mutations (Y111L and Y661D) were developed by prolonged exposure to the highly potent EZH2 enzymatic inhibitor El1 in resistant cells (Gibaja *et al.*, 2016). Considering that Y111L was found on the EZH2 wild-type allele and Y661D was found *cis* to the EZH2 Y641N allele, we investigated the regulation of Y111L and Y641N/Y661D EZH2 protein stability by USP7. These two therapy-resistant EZH2 mutants were found to interact with USP7 (Figure 3E and Figure S6). As with the GOF EZH2 mutants, the protein levels of the therapy-resistant EZH2 mutants were increased by USP7 overexpression (Figure 3F), whereas USP7-knockdown and P5091 treatment destabilized the therapy-resistant EZH2 mutants (Figure 3G and H). Thus, USP7 is implicated in the stabilization of wild-type and oncogenic EZH2 mutant proteins. These results suggest that USP7-specific inhibitors could be promising therapeutic agents for the treatment of EZH2-dependent malignancies.

### USP7 promotes cell migration, invasion, and sphere formation in part through EZH2 stabilization

**Figure 4:**
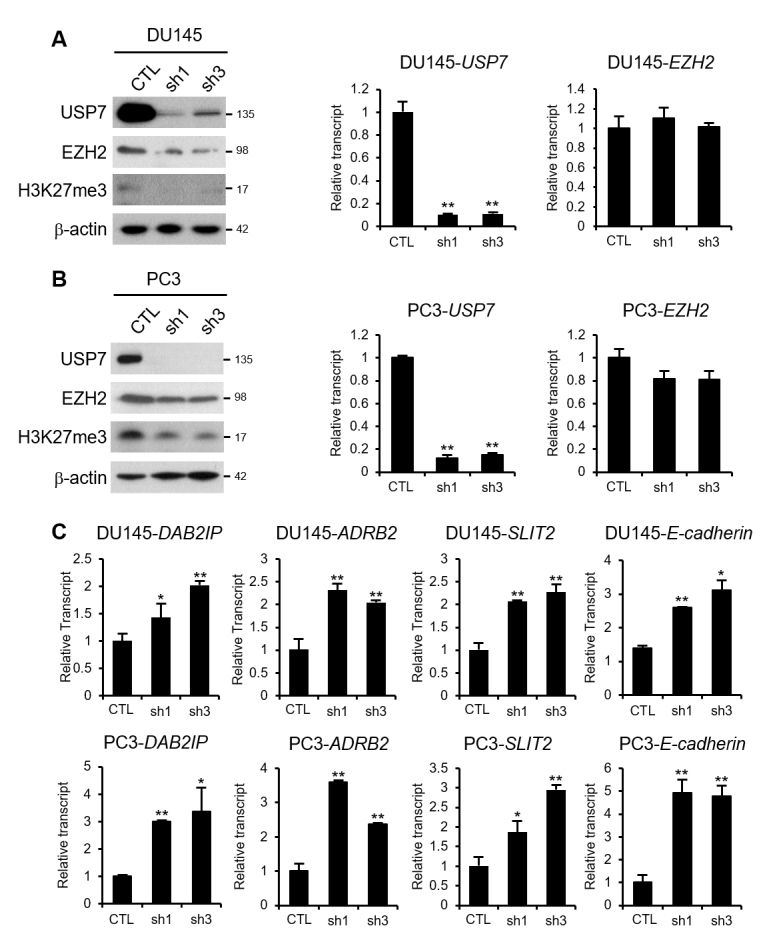
EZH2-repressive target genes are derepressed by USP7-knockdown. (A) Western blot analysis of USP7, EZH2, and H3K27me3 levels in USP7-knockdown DU145 cells. Real-time RT-PCR analysis of the transcript levels of *USP7* and *EZH2* in USP7-knockdown DU145 cells. (B) Western blot analysis of USP7, EZH2, and H3K27me3 levels in USP7-knockdown PC3 cells. Real-time RT-PCR analysis of the transcript levels of *USP7* and *EZH2* in USP7-knockdown PC3 cells. (C) Real-time RT-PCR analysis of the transcript levels of *DAB2IP*, *ADRB2*, *SLIT2*, and *E-cadherin* in USP7-knockdown DU145 or PC3 cells. Values are expressed as the mean ± SD of three independent experiments. The *p* value was obtained by Student’s *t*-test. *p < 0.05, **p < 0.01.

**Figure 5:**
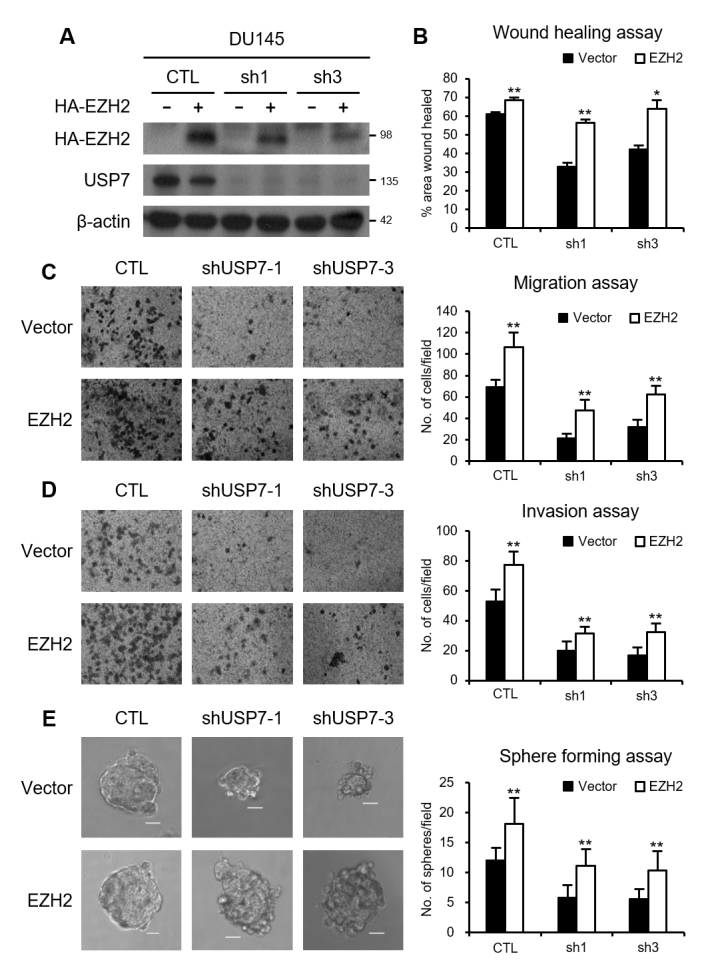
USP7 promotes the migration and invasion of DU145 cells via EZH2 stabilization. (A) HA-EZH2 was expressed in USP7-knockdown DU145 scells and cell lysates were immunoblotted with anti-HA or anti-USP7 antibody. (B) Wound healing assays of USP7-knockdown DU145 stable cells after either vector or EZH2 overexpression. (C) Migration assays of USP7-knockdown DU145 stable cells after either vector or EZH2 overexpression. (D) Matrigel invasion assays of USP7-knockdown DU145 stable cells after either vector or EZH2 overexpression. (E) Sphere formation assays of USP7-knockdown DU145 stable cells after either vector or EZH2 overexpression. The figure shows representative images from each cell, and the scale bar corresponds to 100 μm. Values are expressed as the mean ± SD of three independent experiments (B–E). The *p* value was obtained by Student’s *t*-test. *p < 0.05, **p < 0.01.

In prostate cancer, USP7 overexpression has been reported to show a direct correlation with tumor aggressiveness (Song *et al.*, 2008). The key role of USP7 in the proliferation of prostate cancer cells was identified using USP7 inhibitors and USP7-knockdown (Chen *et al.*, 2015; Morra *et al.*, 2017). As expected in line with the previously reported tumor-promoting ability of USP7, we confirmed the decrease in cell migration, invasion, and sphere-forming abilities in USP7-knockdown PC3 and DU145 stable cell lines (Figure S2). Next, we characterized the physiological function of EZH2 protein stabilization by USP7 in prostate cancer. In USP7-knockdown cell lines, the EZH2 protein and the level of its catalytic target H3K27me3 were decreased (Figure 4A and B). However, no change in the *EZH2* mRNA levels was detected in USP7-knockdown cells, indicating that the regulation of EZH2 by USP7 occurred at the protein level. In prostate cancer, the tumor-promoting effect of EZH2 is attributed to its inhibitory action on the transcription of multiple genes, including *DAB2IP*, *ADRB2*, *SLIT2*, and *E-cadherin* (Yu *et al.*, 2007; Min *et al.*, 2010; Yu *et al.*, 2010). We examined the effects of USP7 on the transcriptional repression function of EZH2. In the USP7-knockdown stable cell lines, the expression of *DAB2IP*, *ADRB2*, *SLIT2*, and *E-cadherin* was derepressed (Figure 4C). Next, we explored whether the decrease in cell migration, invasion, and sphere-forming abilities by USP7-knockdown is attributed to a decrease in EZH2 protein level. We ectopically introduced HA-tagged EZH2 protein in USP7-knockdown cell lines (Figure 5A and Figure S3A). Cell migration, invasion, and sphere-forming activities were restored by EZH2 overexpression in USP7-knockdown prostate cancer cells (Figure 5B-E and Figure S3B-E). Collectively, these results indicate that the promotion of cell migration, invasion, and sphere formation by USP7 is partly attributable to the stabilization of EZH2 in prostate cancer cells.

### USP7 inhibitor P5091 enhances the suppressive activity of EZH2 inhibitors on cell migration and invasion in prostate cancer cells

**Figure 6:**
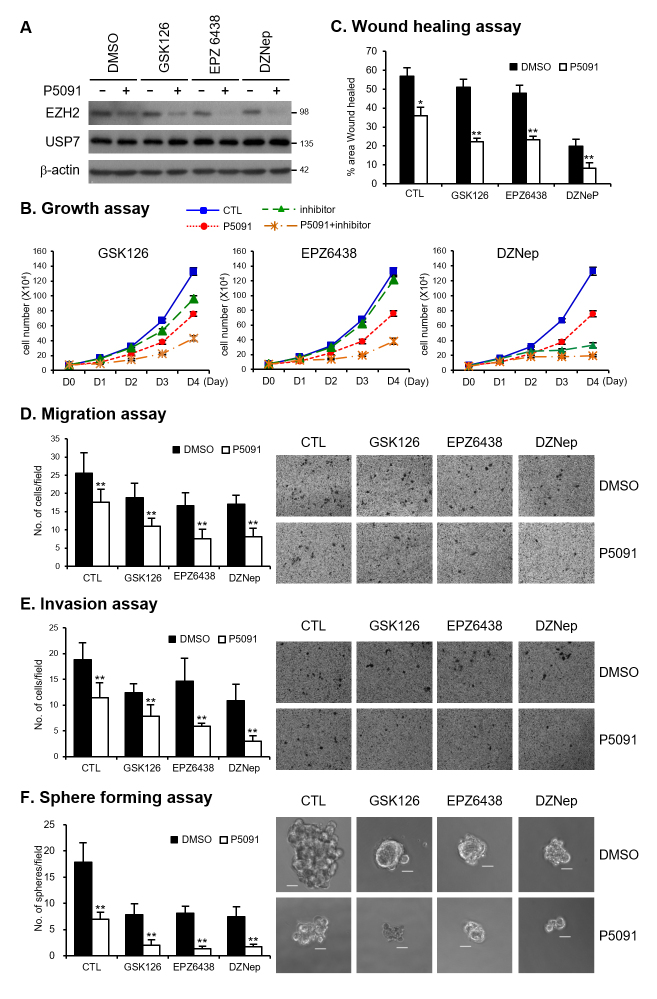
Simultaneous treatment with P5091 and EZH2 inhibitor induces synergistic effects in DU145. (A) DU145 cells were treated with GSK126 (5 μM), EPZ6438 (20 μM), and DZNep (0.5 μM) in the absence or presence of P5091 (2.5 μM) for 48 h. Cell lysates were immunoblotted with anti-EZH2 or anti-USP7 antibody. (B) Growth curves of DU145 cells treated with GSK126 (5 μM), EPZ6438 (10 μM), and DZNep (0.5 μM) in the absence or presence of P5091 (2.5 μM), as indicated. Viable cells were counted by trypan blue-exclusion assay every 24 h after cell seeding. (C) Wound healing assays of DU145 cells treated with GSK126 (5 μM), EPZ6438 (10 μM), and DZNep (0.5 μM) in the absence or presence of P5091 (2.5 μM), as indicated. (D) Migration assays of DU145 cells treated with GSK126 (5 μM), EPZ6438 (20 μM), and DZNep (0.5 μM) in the absence or presence of P5091 (2.5 μM), as indicated. (E) Matrigel invasion assays of DU145 cells treated with GSK126 (5 μM), EPZ6438 (20 μM), and DZNep (0.5 μM) in the absence or presence of P5091 (2.5 μM), as indicated. (F) Sphere formation assays of DU145 cells treated with GSK126 (5 μM), EPZ6438 (20 μM), and DZNep (0.5 μM) in the absence or presence of P5091 (2.5 μM), as indicated. The figure shows representative images from each cell, and the scale bar is 100 μm. Values are expressed as the mean ± SD of three independent experiments (C–F). The *p* value was obtained by Student’s *t*-test. *p < 0.05, **p < 0.01.

Owing to their involvement in cancer development across multiple tumors, USP7 and EZH2 have been considered attractive therapeutic targets and several inhibitors have been developed for each protein. USP7-specific inhibitor P5091 and EZH2 inhibitors have been reported to decrease tumorigenic abilities in several tumor cells (Gulati *et al.*, 2018; Zhou *et al.*, 2018). However, so far, no attempt has been made to investigate the changes in the migratory and invasive potential *in vitro* by simultaneously treating cells with USP7-specific inhibitor and EZH2 inhibitor. We speculated that the reduction of EZH2 protein by USP7 inhibitor could modulate the sensitivity of EZH2 inhibitors in prostate cancer cells. Thus, we tested the sensitivity of prostate cancer cells to EZH2 inhibitors in combination with the USP7-specific inhibitor P5091. We tested three EZH2 inhibitors: GSK126, EPZ6438, and DZNep. DZNep, a *S*-adenosyl-L-homocysteine hydrolase inhibitor, is a first-generation EZH2 inhibitor that has been found to be non-specific toward other methyltransferases (Miranda *et al.*, 2009). GSK126 and EPZ6438 are highly selective *S*-adenosyl-L-methionine competitive small molecule inhibitors of WT and GOF mutant EZH2 methyltransferase activity (McCabe *et al.*, 2012b; Knutson *et al.*, 2014). After treating prostate cancer cells with each EZH2 inhibitor in combination with or without P5091, we assessed the cell proliferation, migration, invasion, and sphere-forming abilities. The protein levels of EZH2 were decreased upon P5091 treatment. EZH2 inhibitors, with or without P5091, did not affect the protein levels of USP7 or EZH2 (Figure 6A and Figure S4A). The growth inhibitory effect of GSK126 and DZNep was enhanced in the presence of P5091 (Figure 6B and Figure S4B). EPZ6438 alone did not show significant growth inhibition at the concentration used in our experiments. Interestingly, compared to P5091 treatment alone, simultaneous treatment with EPZ6438 and P5091 enhanced growth inhibition (Figure 6B and Figure S4B). The inhibitory action of EZH2 inhibitors on wound healing, cell migration, invasion, and sphere formation abilities was augmented in the presence of P5091 (Figure 6C-F and Figure S4C-F). Collectively, our results demonstrated the synergistic inhibition of cell proliferation, migration, invasion, and sphere-forming abilities by combined treatment with P5091 and EZH2 inhibitor. The oncogenic role of EZH2 is diverse and complex. The tumor-promoting activity of EZH2 is attributed not only to its catalytic activity but also to the non-catalytic role of EZH2, which has also been identified (Kim *et al.*, 2015). Therefore, the combination of EZH2 destabilization by P5091 and EZH2 enzyme activity inhibition by an EZH2 inhibitor could be a novel therapeutic strategy for EZH2-dependent cancers.

## Discussion

The physical interaction of USP7 with EZH2 was already identified by de Bie *et al.* (2010), and the possibilities of USP7 function in the regulation of EZH2 ubiquitination status was suggested (de Bie *et al.*, 2010). Recently, Su *et al.* (2019)) reported the stabilization of EZH2 by USP7-mediated deubiquitination in melanoma cells. However, the role of USP7 in the modulation of EZH2 protein stability in prostate cancer cells has not yet been elucidated. Herein, we identified that EZH2 is a deubiquitination target of USP7 and that EZH2 protein stability is increased by USP7. Destabilization of EZH2 protein by USP7-knockdown decreased the transcriptional repression activity of EZH2 in prostate cancer cells. Furthermore, we found that the promotion of cell migration, invasion, and sphere-forming potential by USP7 was partly dictated by EZH2 protein stabilization. Notably, combined treatment with USP7 inhibitor P5091 and EZH2 inhibitors (GSK126, EPZ6438, and DZNep) augmented the inhibition of cell migration, invasion, and sphere-forming abilities in prostate cancer cells.

As EZH2 is an epigenetic regulator important for cancer development and progression across various tumors, the abundance and activity of EZH2 should be finely controlled depending on the various signals and pathophysiological conditions of cells. Dysregulation of EZH2, including its overexpression and mutational changes, is observed with high frequency in multiple cancers (Varambally *et al.*, 2002; Hu *et al.*, 2010; Guo *et al.*, 2016). This has led to the development of small molecule inhibitors targeting the enzymatic activity of EZH2 (Gulati *et al.*, 2018). However, the oncogenic function of EZH2 is complex and diverse. The tumor-promoting activity of EZH2 in castration-resistant prostate cancer is mediated by its activity as a coactivator for AR (Xu *et al.*, 2012). Kim *et al.* (2015) demonstrated the non-enzymatic contribution of EZH2 to PRC2 complex stabilization in SWI/SNF-mutant cancers (Kim *et al.*, 2015). These results raise the concern that EZH2 enzymatic inhibitors alone may not completely inhibit the carcinogenic activity of EZH2. In addition, multiple acquired mutations that confer resistance to EZH2 inhibitors, including GSK126, EPZ6438, and EI1, have been identified in DLBCL cell line models ((Gibaja *et al.*, 2016; Bisserier and Wajapeyee, 2018). As an alternative, EZH2 suppression along with other targeted agents is recommended to enhance treatment efficacy and minimize the development of resistance. Various trials using EZH2 inhibitors in combination with conventional chemotherapy or other targeted agents are currently in progress (Gulati *et al.*, 2018). However, no combination treatment with an agent targeting the EZH2 protein stability modulation pathway has been attempted. In this report, we showed the synergistic inhibition of cell proliferation, migration, invasion, and sphere-forming abilities by combined treatment with USP7 inhibitor P5091 and EZH2 inhibitors.

The UPS dynamically regulates the protein stability of EZH2. Therefore, UPS modulators that are involved in EZH2 protein stability could be adopted as therapeutic agents that act by altering EZH2 abundance in the cell. Several E3 ubiquitin ligases have been reported for ubiquitin-mediated EZH2 degradation (Wang *et al.*, 2018). In the reverse process, DUBs, including USP21 and ZRANB1, deubiquitinate and increase the protein stability of EZH2 (Chen *et al.*, 2017; Zhang *et al.*, 2018). We previously reported the deubiquitinating activity of USP44 and subsequent EZH2 protein stabilization in prostate cancer cells and suggested that targeting USP44 might be an efficient anticancer strategy for EZH2-dependent cancers irrespective of the enzymatic activity of EZH2 (Park *et al.*, 2019). However, because of the lack of currently available USP44-specific inhibitors, we could not verify our hypothesis. Here, we investigated USP7 as an EZH2 stabilizer in prostate cancer cells. USP7 and USP44 stabilized both wild-type and oncogenic mutants of EZH2. However, further studies are needed to clarify the differences in the physiological function and mechanisms of USP7 and USP44 as EZH2 protein stabilizers in prostate cancer. In addition, the immunohistochemical analysis of USP7 (or USP44) and EZH2 in a series of primary prostatic biopsies is required to examine whether a correlation between their expression levels can be detected. These analyses may help to sort out the expression patterns, which may improve the tumor classification and provide therapeutic indications.

USP7 has been regarded as a promising target of anticancer therapies because it regulates the stability of several proteins associated with cancer progression; findings on the potential of USP7 inhibitors as agents for prostate cancer treatment have already been reported. Morra *et al.* (2017) demonstrated the synergistic effects of combined treatment with USP7 inhibitors and PARP inhibitors in hormone-sensitive and castration-resistant prostate cancer cells (Morra *et al.*, 2017). Pharmacological inhibition of USP7 downregulates the expression of CCDC6 involved in homologous recombination and results in DNA repair defects that increase the sensitivity of PARP inhibitors (Leone *et al.*, 2015). Our study showed the synergistic effects of P5091 and EZH2 inhibitors based on the regulation of EZH2 stabilization by USP7 in prostate cancer cells. Therefore, in the future, it may be possible to combine these drugs or apply them sequentially depending on the expression patterns of USP7, CCDC6, and EZH2 in individual cases of prostate cancer to provide novel forms of customized therapy.

## Supplementary Material

The following online material is available for this article:

Figure S1 Interaction between USP7 and several histone-modifying enzymes.

Figure S2 USP7-knockdown decreases the cell migration, invasion, and sphere-forming abilities in DU145 and PC3 cells.

Figure S3 USP7 promotes the migration and invasion of PC3 cells via EZH2 stabilization.

Figure S4 Simultaneous treatment with P5091 and EZH2 inhibitor induces synergistic effects in PC3.

Figure S5 Sequencing chromatograms of USP7 constructions.

Figure S6 Sequencing chromatograms of EZH2 mutants.
