# Supplementary material for: USP7 deubiquitinates and stabilizes EZH2 in prostate cancer cells
Source: Genet Mol Biol. 2020 May 20;43(2):e20190338. doi: 10.1590/1678-4685-GMB-2019-0338 (PMC7252518; doi:10.1590/1678-4685-GMB-2019-0338)
Supplement: Figure S1 [file 1415-4757-GMB-43-2-e20190338-s1.pdf]

Supplementary Material to "USP7 deubiquitinates and stabilizes EZH2 in prostate cancer cells"

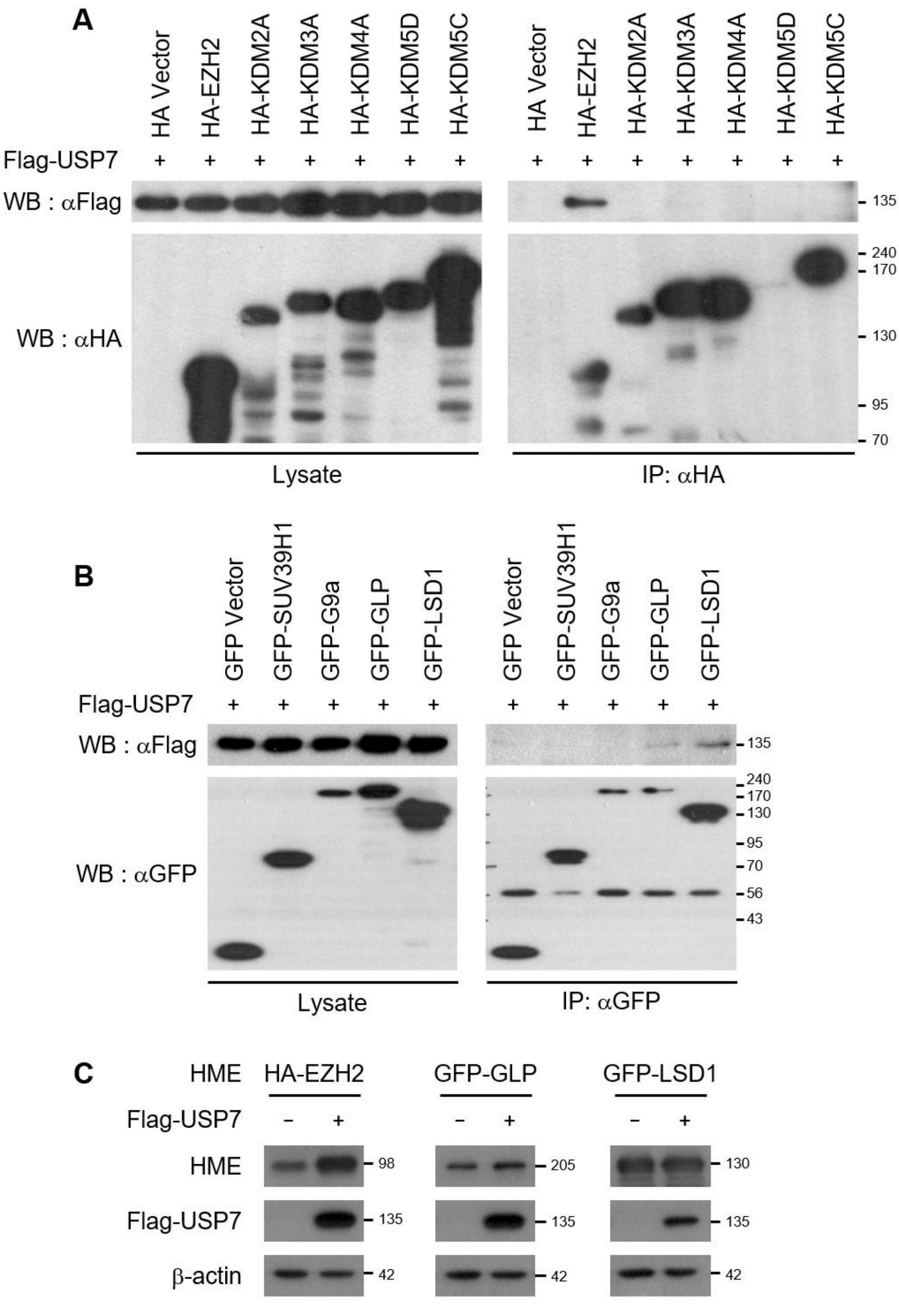

Figure S1 - Interaction between USP7 and several histone-modifying enzymes. (A) HEK293T cells were transfected as indicated. Cell lysates were immunoprecipitated with HA antibody followed by immunoblotting. (B) HEK293T cells were transfected as indicated. Cell lysates were immunoprecipitated with GFP antibody followed by immunoblotting. (C) HEK293T cells were transfected as indicated. Protein levels of HME and Flag-USP7 were verified by immunoblotting. *HME: histone-modifying enzymes.
