# Supplementary material for: USP7 deubiquitinates and stabilizes EZH2 in prostate cancer cells
Source: Genet Mol Biol. 2020 May 20;43(2):e20190338. doi: 10.1590/1678-4685-GMB-2019-0338 (PMC7252518; doi:10.1590/1678-4685-GMB-2019-0338)
Supplement: Figure S2 [file 1415-4757-GMB-43-2-e20190338-s2.pdf]

Supplementary Material to “USP7 deubiquitinates and stabilizes EZH2 in prostate cancer cells”

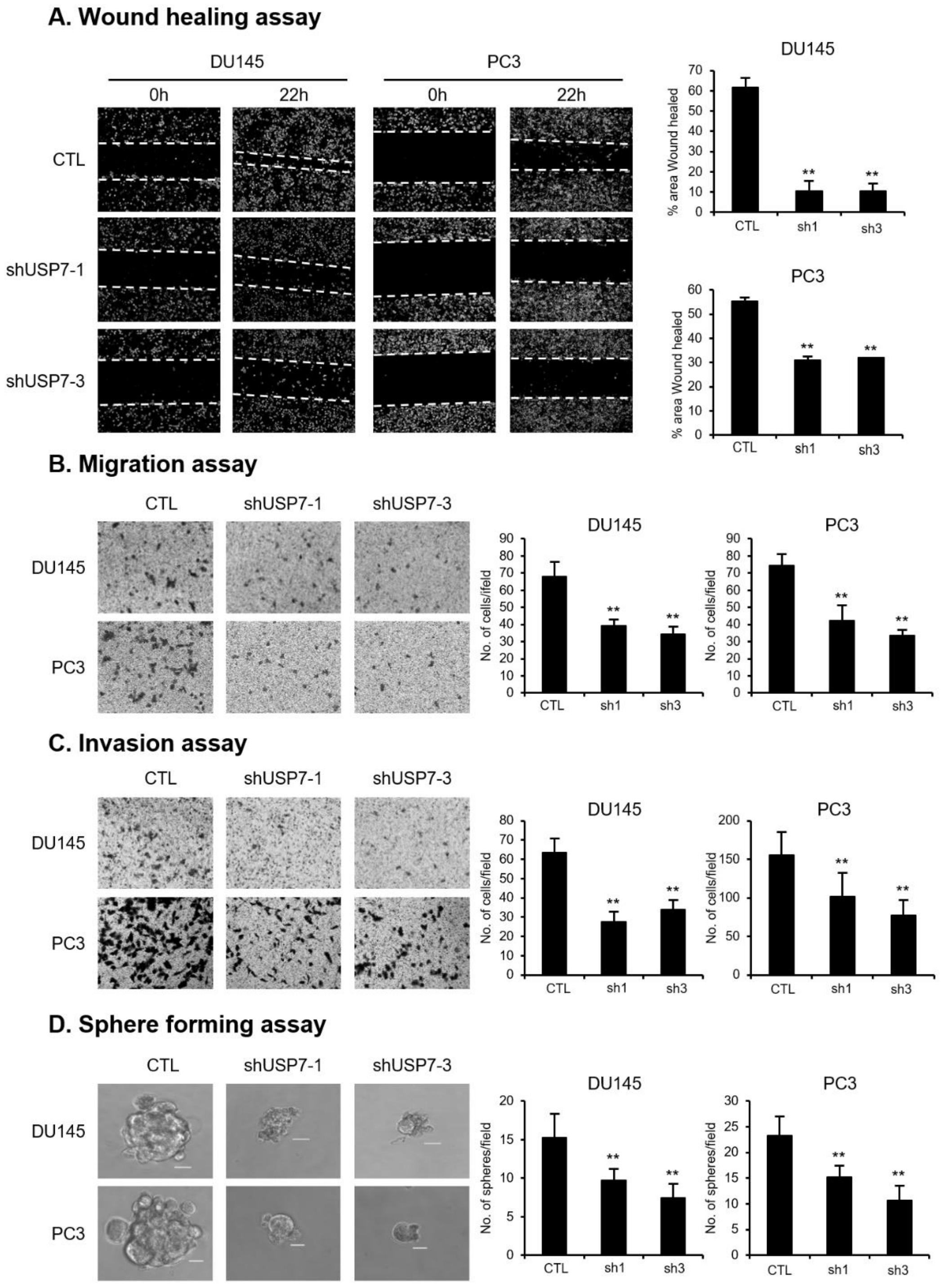

Figure S2 - USP7-knockdown decreases the cell migration, invasion, and sphere-forming abilities in DU145 and PC3 cells. (A) Wound healing assays of USP7-knockdown stable cell lines. (B) Migration assays of USP7-knockdown stable cell lines. (C) Matrigel invasion assays of USP7-knockdown stable cell lines. (D) Sphere formation assays of the USP7-knockdown stable cell lines. The figure shows the representative images from each cell and the scale bar is 100 μm. Values are expressed as the mean ± SD of three independent experiments (A–D). The *p* value was obtained by Student’s *t*-test. *$p < 0.05$, **$p < 0.01$.
