# Supplementary material for: USP7 deubiquitinates and stabilizes EZH2 in prostate cancer cells
Source: Genet Mol Biol. 2020 May 20;43(2):e20190338. doi: 10.1590/1678-4685-GMB-2019-0338 (PMC7252518; doi:10.1590/1678-4685-GMB-2019-0338)
Supplement: Figure S3 [file 1415-4757-GMB-43-2-e20190338-s3.pdf]

Supplementary Material to "USP7 deubiquitinates and stabilizes EZH2 in prostate cancer cells"

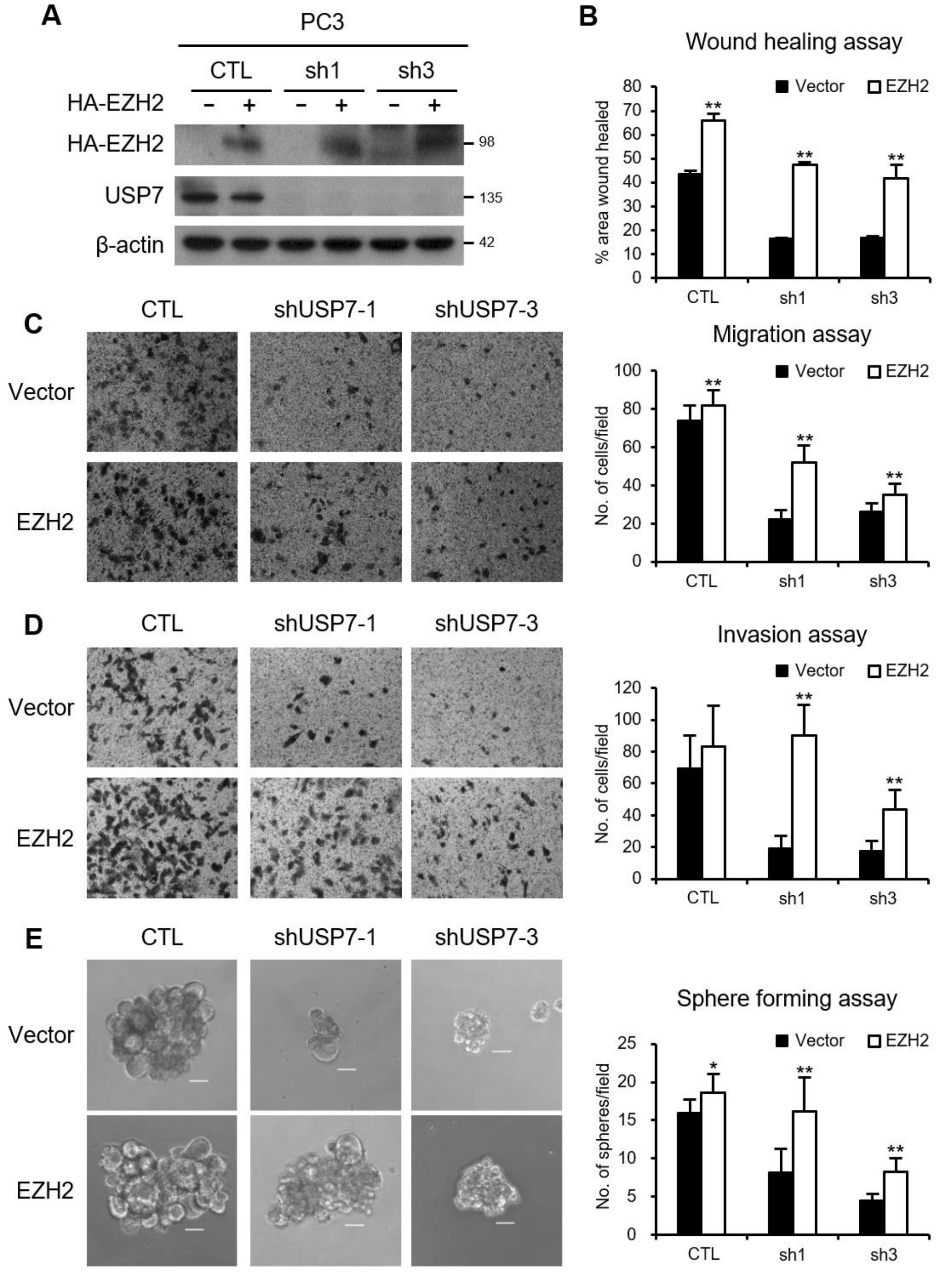

Figure S3 - USP7 promotes the migration and invasion of PC3 cells via EZH2 stabilization. (A) HA-EZH2 was expressed in USP7-knockdown PC3 stable cells and cell lysates were immunoblotted with anti-HA or anti-USP7 antibody. (B) Wound healing assays of USP7-knockdown PC3 stable cells after either vector or EZH2 overexpression. (C) Migration assays of USP7-knockdown PC3 stable cells after either vector or EZH2 overexpression. (D) Matrigel invasion assays of USP7-knockdown PC3 stable cells after either vector or EZH2 overexpression. (E) Sphere formation assays of USP7-knockdown PC3 stable cells after either vector or EZH2 overexpression. The figure shows representative images from each cell, and the scale bar corresponds to 100 μm. Values are expressed as the mean ± SD of three independent experiments (B–E). The p value was obtained by Student's t-test. *$p < 0.05$, **$p < 0.01$.
