# Supplementary material for: USP7 deubiquitinates and stabilizes EZH2 in prostate cancer cells
Source: Genet Mol Biol. 2020 May 20;43(2):e20190338. doi: 10.1590/1678-4685-GMB-2019-0338 (PMC7252518; doi:10.1590/1678-4685-GMB-2019-0338)
Supplement: Figure S4 [file 1415-4757-GMB-43-2-e20190338-s4.pdf]

Supplementary Material to "USP7 deubiquitinates and stabilizes EZH2 in prostate cancer cells"

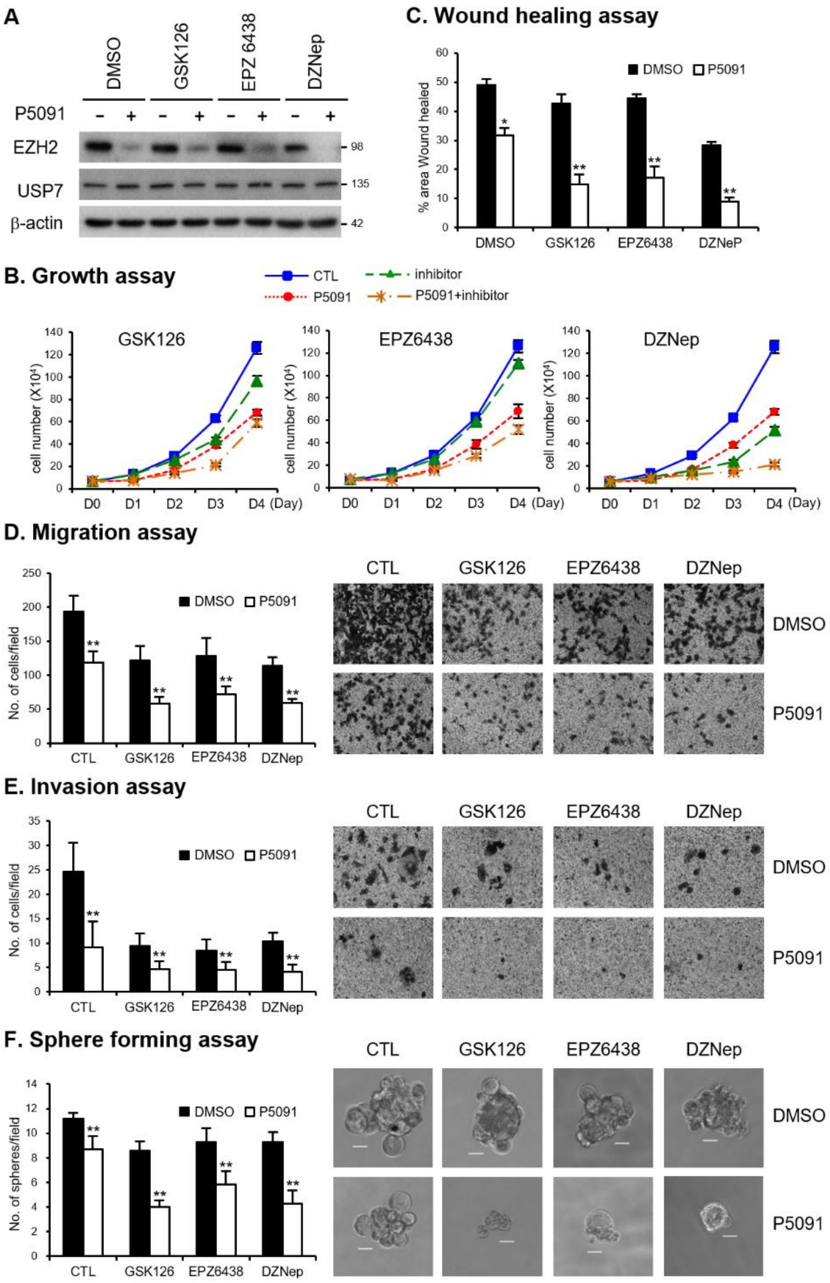

Figure S4 - Simultaneous treatment with P5091 and EZH2 inhibitor induces synergistic effects in PC3. (A) PC3 cells were treated with GSK126 (5 μM), EPZ6438 (20 μM), and DZNep (0.5 μM) in the absence or presence of P5091 (2.5 μM) for 48 h. Cell lysates were immunoblotted with anti-EZH2 or anti-USP7 antibody. (B) Growth curves of PC3 cells treated with GSK126 (5 μM), EPZ6438 (10 μM), and DZNep (0.5 μM) in the absence or presence of P5091 (2.5 μM), as indicated. Viable cells were counted by trypan blue-exclusion assay every 24 h after cell seeding. (C) Wound healing assays of PC3 cells treated with GSK126 (5 μM), EPZ6438 (10 μM), and DZNep (0.5 μM) in the absence or presence of P5091 (2.5 μM), as indicated. (D) Migration assays of PC3 cells treated with GSK126 (5 μM), EPZ6438 (20 μM), and DZNep (0.5 μM) in the absence or presence of P5091 (2.5 μM), as indicated. (E) Matrigel invasion assays of PC3 cells treated with GSK126 (5 μM), EPZ6438 (20 μM), and DZNep (0.5 μM) in the absence or presence of P5091 (2.5 μM), as indicated. (F) Sphere formation assays of PC3 cells treated with GSK126 (5 μM), EPZ6438 (20 μM), and DZNep (0.5 μM) in the absence or presence of P5091 (2.5 μM), as indicated. The figure shows representative images from each cell, and the scale bar is 100 μm. Values are expressed as the mean ± SD of three independent experiments (C–F). The *p* value was obtained by Student's *t*-test. *$p < 0.05$, **$p < 0.01$.
