# Supplementary material for: USP7 deubiquitinates and stabilizes EZH2 in prostate cancer cells
Source: Genet Mol Biol. 2020 May 20;43(2):e20190338. doi: 10.1590/1678-4685-GMB-2019-0338 (PMC7252518; doi:10.1590/1678-4685-GMB-2019-0338)
Supplement: Figure S5 [file 1415-4757-GMB-43-2-e20190338-s5.pdf]

Supplementary Material to "USP7 deubiquitinates and stabilizes EZH2 in prostate cancer cells"

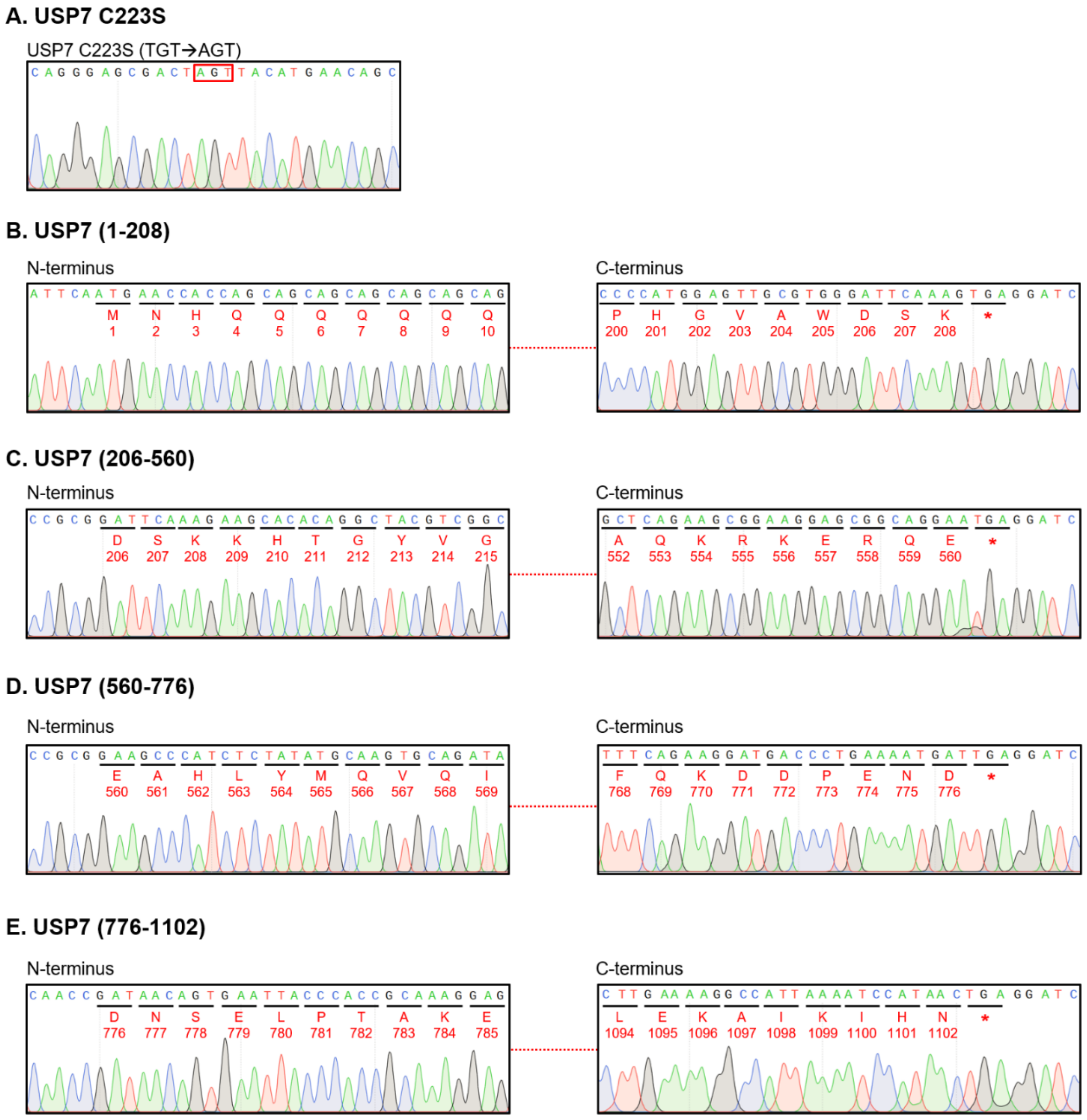

Figure S5 - Sequencing chromatograms of USP7 constructions: (A) USP7 C223S, (B) USP7 (1-208), (C) USP7 (206-560), (D) USP7 (560-776), (E) USP7 (776-1102). Mutated codon is highlighted with red box (A). Several amino acids at the N- and C-terminus of the deletion mutant are indicated in red (B-E). Stop codon is denoted with *.
