# Supplementary material for: USP7 deubiquitinates and stabilizes EZH2 in prostate cancer cells
Source: Genet Mol Biol. 2020 May 20;43(2):e20190338. doi: 10.1590/1678-4685-GMB-2019-0338 (PMC7252518; doi:10.1590/1678-4685-GMB-2019-0338)
Supplement: Figure S6 [file 1415-4757-GMB-43-2-e20190338-s6.pdf]

Supplementary Material to "USP7 deubiquitinates and stabilizes EZH2 in prostate cancer cells"

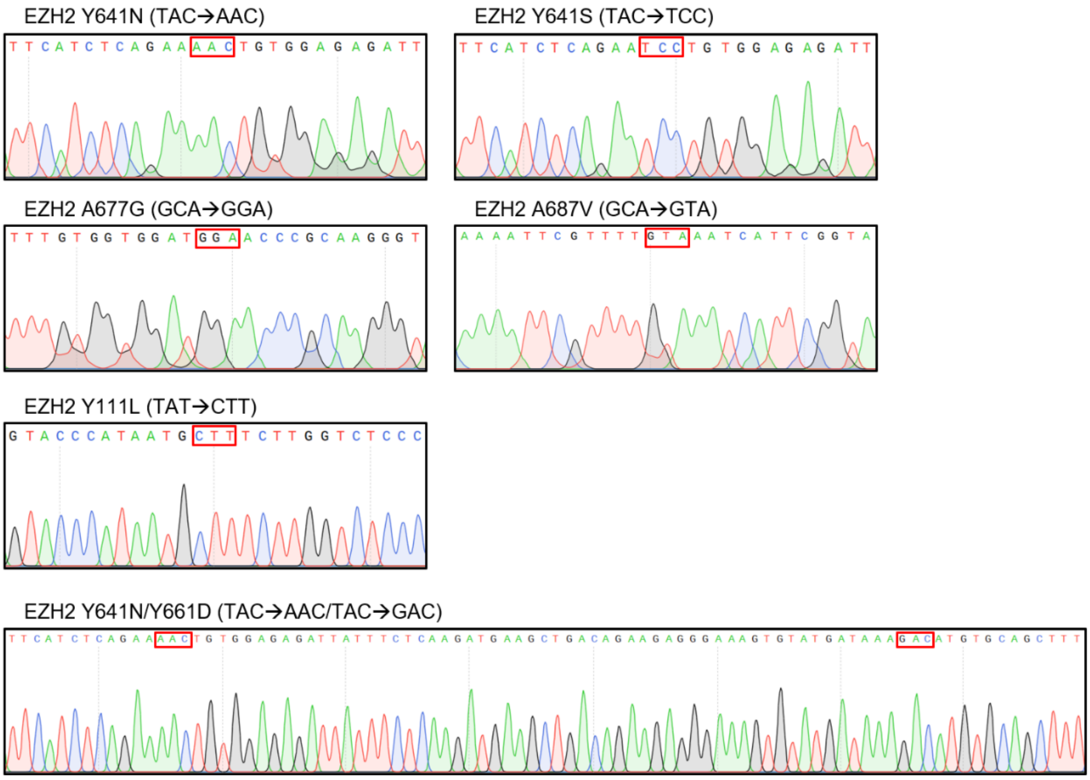

Figure S6 - Sequencing chromatograms of EZH2 mutants: EZH2 Y641N (TAC→AAC), EZH2 Y641S (TAC→TCC), EZH2 A677G (GCA→GGA), EZH2 A687V (GCA→GTA), EZH2 Y111L (TAT→CTT), and EZH2 Y641N/Y661D (TAC→AAC, TAC→GAC). Mutated codons are highlighted with red box.
